# Identification of Variable Traits among the Methicillin Resistant and Sensitive Coagulase Negative Staphylococci in Milk Samples from Mastitic Cows in India

**DOI:** 10.3389/fmicb.2017.01446

**Published:** 2017-07-31

**Authors:** Sudipta Mahato, Hiral U. Mistry, Shalini Chakraborty, Paresh Sharma, R. Saravanan, Vasundhra Bhandari

**Affiliations:** ^1^National Institute of Animal Biotechnology Hyderabad, India; ^2^Department of Animal Genetics and Breeding, Veterinary College and Research Institute Namakkal, India; ^3^School of Life Sciences, University of Hyderabad Hyderabad, India

**Keywords:** coagulase negative staphylococci, bovine mastitis, antibiotic susceptibility, OS-MRS, *pvl* gene

## Abstract

Methicillin resistant *Staphylococcus aureus* causing bovine mastitis has been very well investigated worldwide. However, there are only limited reports on the characterization of methicillin resistant and sensitive coagulase negative staphylococci (CoNS) across the globe. Hence, in the present study, we aim to determine the phenotypic traits based on antimicrobial susceptibility profile and genotypic characterization by verifying the presence of resistance determinants, virulence and toxin genes present in the CoNS causing clinical mastitis. We obtained 62 CoNS isolates from 167 mastitic milk samples collected from three different states of India. The 62 isolates comprises of 10 different CoNS species *S. sciuri*, *S. haemolyticus, S. chromogenes*, *S. saprophyticus*, *S. xylosu*s, *S. simulans*, *S. agnetis*, *S. epidermidis*, *S. gallinarum*, and *S. cohinii*. Susceptibility screening against 11 antibiotics determined 45.16% isolates as multidrug resistant (resistant to more than two class of antibiotic), 46.74% resistant (one or two antibiotic class) and 8.06% isolates were pan-sensitive (sensitive to all drugs). High resistance was observed against oxacillin and cefoxitin, whereas all isolates were susceptible toward vancomycin and linezolid. Fifty three isolates were methicillin resistant and 9 isolates were sensitive as determined by oxacillin susceptibility assay. The methicillin resistance gene, *mecA* was found in 95.16% isolates and staphylococcal cassette chromosome *mec* (SCC*mec*) typing predominantly revealed Type III (*n* = 34) and Type V (*n* = 18). Interestingly, 11.9% of *mecA* positive isolates were oxacillin susceptible and referred as oxacillin susceptible *mecA* positive staphylococci (OS-MRS). Additionally, genes encoding for enterotoxin, (*sea, seb, seh, see*) toxic shock syndrome (*tsst*), exfoliatin (*eta, etb, etd*) and virulence (*pvl, Y-hlg*) were also screened. Of all the genes examined, 67.74% of isolate were positive for the *Y-hlg* gene, followed by the *sea* gene in 25.8% whereas in none of the isolates the *eta* and the *etb* gene was amplified. The study also highlights the incidence of clinical isolates of CoNS, which are harboring the toxin and the virulence genes rendering them as a more potential threat. This is the first report of animal origin OS-MRS from India, which emphasizes on the inclusion of both the genetic and phenotypic test for proper characterization of CoNS and preventing resistant strain misidentification.

## Introduction

Coagulase negative staphylococci (CoNS) belong to the group of opportunistic pathogen causing infection in humans and animals. In humans, they are associated with endocarditis, septicemia and blood stream infection, etc., whereas in dairy cattle they mainly cause inflammation of the udder resulting in bovine mastitis (Becker et al., 2014). There are two major forms of mastitis in dairy cattle, clinical and sub-clinical mastitis. In clinical mastitis, the symptoms like swelling, redness or hardness of udder, decrease in milk yield with appearance of clots, flakes, pus or change in milk color, etc., are clearly observed, which makes the diagnosis easy. While in sub-clinical mastitis cases, the symptoms are invisible and hence, difficult to diagnose and results in major cost implications. Mastitis greatly affects the animal health negatively impacting the economy of countries like India, which is one of the largest producers of milk in the world (Preethirani et al., 2015). Bovine mastitis is mainly caused by the group of bacterial species belonging to *Streptococcus* sp. *Escherichia coli*, and *Staphylococci* sp., etc. (Krishnamoorthy et al., 2017). CoNS were earlier considered as the minor group of bacteria with less pathogenicity, as they were only reported in sub-clinical mastitis cases and hence, less attention was paid toward them (Pyörälä and Taponen, 2009). However, reports of clinical mastitis infection caused by various CoNS species have now surfaced largely and they have emerged as an important pathogen (Pyörälä and Taponen, 2009; Preethirani et al., 2015; Srednik et al., 2015).

Infections caused by methicillin resistant staphylococci (MRS) are more harmful due to prolonged treatment duration and limited drug options. Recent reports from different parts of the world revealed MRS as an emerging cause of infection and potent threat to the dairy industry and public health due to its zoonotic potential (Luthje and Schwarz, 2006; Walther and Perreten, 2007; Moodley and Guardabassi, 2009; Weiβ et al., 2013). However, methicillin resistant *Staphylococcus aureus* (MRSA) have been more widely studied and well characterized in comparison to MRS especially in India (Monecke et al., 2007; Fessler et al., 2010; Türkyilmaz et al., 2010). Increased drug exposure and use of antibiotics in animal diseases also poses a hazard to human health whose impact and effect is still not well characterized but may result in emergence of antibiotic resistant strains making the surveillance of antibiotic susceptibility very crucial (Pyörälä and Taponen, 2009; Fessler et al., 2010; Preethirani et al., 2015; Srednik et al., 2015). Another, concern associated with CoNS is that they may also act as reservoirs of antimicrobial resistance genes (Becker et al., 2014), and can transfer the resistance gene into the *S. aureus* genome leading to the development of new and multidrug resistant strains (Otto, 2012; Vitali et al., 2014).

Therefore, susceptibility profiling of the CoNS clinical isolates will provide us with valuable information regarding the efficacy of the antibiotics used in the field and warn us about the emergence of antibiotic resistant strain. Overall, the knowledge will be helpful to veterinarians for designing an effective treatment regime and to policy makers, which can formulate a better strategy to control bovine mastitis. Another aspect of CoNS biology, which is understudied, is the evaluation of the toxin genes in the clinical isolates. Recently, the presence of toxin genes in CoNS strains has been reported, which may be responsible for food poisoning, especially the enterotoxin genes which are heat stable and may not get destroyed after boiling of milk or proper cooking of dairy products and meat (Veras et al., 2008).

A very little information is available on the CoNS isolates in the context of phenotypic and genotypic traits correlation of MRS, their response against other antimicrobials and the prevalence of virulence or toxin genes. Therefore, the present study aims to molecularly and phenotypically characterize CoNS causing bovine mastitis in India.

## Materials and Methods

### Isolation and Characterization of CoNS Isolates

Sixty-two CoNS isolates have been isolated from 167 milk samples of cows suffering from clinical bovine mastitis belonging to different farms (organized and unorganized) in three different states of India, Telangana (*n* = 78), Andhra Pradesh (*n* = 50) and Tamil Nadu (*n* = 39). Before milk collection, oral consent was obtained from the farm owners and the professional veterinarian collected samples. Milk samples were cultured in trypticase soy broth (TSB, Himedia, Mumbai, India) and incubated overnight at 37°C, further, the broth was streaked on mannitol salt agar plate. Pinkish-white colonies on the mannitol salt agar plate (Himedia, Mumbai, India) were presumed to be CoNS. Further, confirmation of the CoNS colonies was done by biochemical test [Gram staining kit (Sigma, St. Louis, MO, United States), and catalase test (BD, New Delhi, India)] followed by sequence analysis of the amplified universal *16S rRNA* PCR product (>1.2 Kb) for species confirmation. Briefly, in order to perform PCR, genomic DNA was extracted from each isolate using Wizard genomic DNA kit (Promega, Madison, WI, United States). Briefly, 2 mL of overnight grown culture in TSB were pelleted at 10,000 × *g* for 3 min. The pellet was then washed thrice with 1 × PBS at 8000 × *g*, 3 min and resuspended in 500 μl of 50 mM EDTA, lysostaphin (100 μg/ml; Sigma, St. Louis, MO, United States) and lysozyme (100 μg/ml; Sigma, St. Louis, MO, United States) for 1 hr at 37°C followed by manufacturer’s instructions. The *16S rRNA* gene PCR was carried out according to the conditions described elsewhere (Srinivasan et al., 2015; Mistry et al., 2016).

### PCR Assay and SCC *mec* Typing

All strains were checked for the presence of *mecA*, *mecC*, and *van A* using PCR as described previously (Stegger et al., 2012). Further, SCC *mec* cassette element classification was done for all *mecA* positive isolates, as described earlier (Zhang et al., 2005).

### Toxin Genes Profiling

Genomic DNA isolated from the CoNS isolates were subjected to amplification of five enterotoxin genes (*sea, seb, sed, see*, and *seh*), three exfoliatin gene (*eta, etb*, and *etd*), toxic shock syndrome gene (*tsst*) and two virulence gene (Panton-Valentine leukocidin gene, *pvl* and Ẏ-hemolysin, *Ý-hlg* gene). All primers and PCR condition used for toxin genes amplification was performed as described previously (Moore and Lindsay, 2001; Xie et al., 2011; Okolie et al., 2015). Additionally, sequencing of few amplified PCR products was carried in order to confirm the toxin genes sequences. List of primers and their sequences are mentioned in **Table 1**.

**Table 1:** Details of primers used in the study.

S. No.	Gene name	Sequence	Amplicon Size	Reference
1	*16S rRNA*	**F**: AGAGTTTGATCCTGGCTCAG	>1.2 Kb	Srinivasan et al., 2015
		**R**:TAC GYT ACC TTG TTA CGA CTT		
2	*mecA*	**F**:TCCAGATTACAACTTCACCAGG	162 bp	Stegger et al., 2012
		**R**:CCACTTCATATCTTGTAACG		
3	*mecC*	**F**:GAAAAAAAGGCTTAGAACGCCTC	138 bp	Stegger et al., 2012
		**R**:GAAGATCTTTTCCGTTTTCAGC		
4	*sea*	**F**: ATTAACCGAAGGTTCTGTAGA	552 bp	Xie et al., 2011
		**R**:TTGCGTAAATCTGAA TT		
5	*seb*	**F**:TGTATGTATGGAGGTGTAAC	270 bp	Xie et al., 2011
		**R**: ATAGTGACGAGTTAGGTA		
6	*sed*	**F**:CTAGTTTGGTAATATCTCCT	317 bp	Xie et al., 2011
		**R**: TAATGCTATATCTTATAGGG		
7	*see*	**F**:TAGATAAAGTTAAAACAAGC	170 bp	Xie et al., 2011
		**R**:TAACTTACCGTGGACCCTTC		
8	*seh*	**F**:CACATCATATGCGAAAGCAGA	617 bp	Xie et al., 2011
		**R:**CCTTTTAAATCATAAATGTCGAATGA		
9	*eta*	**F:**ACTGTAGGAGCTAGTGCATTTGT	190 bp	Xie et al., 2011
		**R**:TGGATACTTTTGTCTATCTTTTTC ATCAAC		
10	*etb*	**F**:CAGATAAAGAGCTTTATA CAC ACATTAC	612 bp	Xie et al., 2011
		**R**:AGTGAACTTATCTTTCTATTG AAAAACACTC		
11	*etd*	**F:**CGCAAATACATATGAAGAATCTGA	452 bp	Xie et al., 2011
		**R:**TGTCACCTTGTTGCAAATCTATAG		
12	*tsst*	**F**:TGCAAAAGCATCTACAAACGA	499 bp	Xie et al., 2011
		**R:**TGTGGATCCGTCATTCATTG		
13	*Y-hlg*	**F:**CCAATCCGTTATTAGAAAATGC	937 bp	Moore and Lindsay, 2001
		**R:**CCATAGACGTAGCAACGGAT		
14	*pvl*	**F:**TTACACAGTTAAATATGAAGTGAACTGGA	118 bp	Okolie et al., 2015
		**R:**AGCAAAAGCAATGCAATTGATG		


### Antimicrobial Susceptibility

Disk diffusion and micro-broth dilution assays were used to determine antimicrobial susceptibility of the 62 clinical isolates as per CLSI guidelines. All the cultures were inoculated into Mueller–Hinton Broth (Himedia, Mumbai, India) and incubated overnight at 37°C. The turbidity of the cultures was adjusted to 0.5 McFarland standard (Himedia, Mumbai, India) and was streaked onto Mueller–Hinton Agar (Himedia, Mumbai, India) plates for disk diffusion while Mueller–Hinton broth was used for micro-broth dilution assay. The disk diffusion assay was done for 8 antibiotics; clindamycin (2 μg), erythromycin (15 μg), gentamicin (10 μg), ciprofloxacin (5 μg), tetracycline (30 μg), rifampicin (5 μg), cefoxitin (30 μg), and teicoplanin (30 μg). All the antibiotic disks were procured from Himedia, Mumbai, India. The MICs against oxacillin (Sigma, St. Louis, MO, United States), vancomycin (Sigma, St. Louis, MO, United States) and linezolid (Sigma, St. Louis, MO, United States) were evaluated by micro-broth dilution method using resazurin dye as described previously (Sarker et al., 2007; Mistry et al., 2016). ATCC 29213 and ATCC 25923 were used as a recommended quality control strains for antibiotic susceptibility assays as per CLSI guidelines (Clinical and Laboratory Standards Institute, 2014).

## Results

### CoNS Species Causing Bovine Mastitis

The 62 clinical isolates obtained belonged to 10 different CoNS species identified were *S. sciuri* (*n* = 20), *S. haemolyticus* (*n* = 13), and *S. chromogenes* (*n* = 10). Few infections were also caused by, *S. saprophyticus* (*n* = 6), *S. xylosu*s (*n* = 5), *S. simulans* (*n* = 2), *S. agnetis* (*n* = 2), *S. epidermidis* (*n* = 2), *S. gallinarum* (*n* = 1), and *S. cohinii* (*n* = 1).

### Antibiotic Resistance Genes PCR and SCC*mec* Classification

PCR of resistance genes *mecA*, *mecC*, and *vanA* were done for all isolates. In none of the isolates, *mecC* and *vanA* were amplified. The *mecA* gene was detected in 95.16% isolates. SCC*mec* classification of those 59 isolates revealed type III (*n* = 34) and type V (*n* = 18) with seven non-typeable strains.

### Toxin Gene Profiling

Toxin and virulence genes were profiled in 62 CoNS isolates. The *sea* gene was amplified in 9.16% isolates belonging to five different species, *S. sciuri* (*n* = 6), *S. haemolyticus* (*n* = 7), *S. xylosus* (*n* = 1), *S. saprophyticus* (*n* = 1), and *S. chromogenes* (*n* = 1). The amplification of the *seh* gene was observed in three *S. chromogenes*, two *S. haemolyticus* followed by one isolate belonging to *S. saprophyticus, S. epidermidis.* Amplification for the *seb* gene was observed in single isolate of *S. sciuri* while in one *S. chromogenes* isolate the see gene was positive.

**FIGURE 1:**
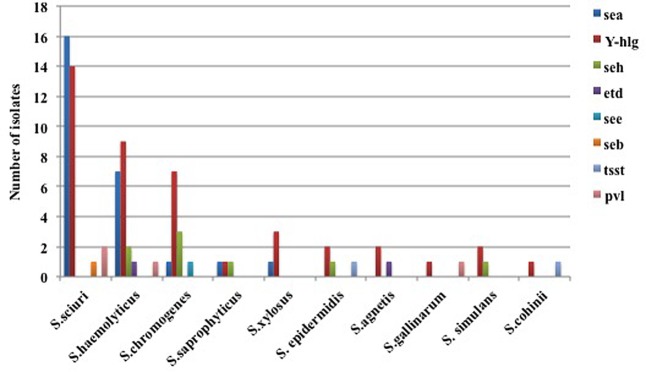
Presence of toxin genes in the clinical isolates of coagulase negative staphylococci (CoNS). The toxin genes *sea*, *seb*, *see*, *seh, etd*, *tsst*, *Ý-hlg*, and *pvl* were screened using PCR. The bar indicates the different species of CoNS showing amplification of respective toxin genes.

Out of the three exfoliatin genes, the *etd* gene was amplified in two isolates (*S. agnetis* and *S. haemolyticus*) while the *eta* and the *etb* were not amplified. The *tsst* gene was amplified in only two isolates belonging to *S. agnetis* and *S. haemolyticus* species. The virulence genes; *Ý-hlg* were positive in 42 clinical isolates belonging to all species with predominance of *S. sciuri* (*n* = 14) and *S. haemolyticus* (*n* = 9) whereas only four isolates, two of *S. sciuri* and one each of *S. haemolyticus* and *S. gallinarum* were positive for the *pvl* gene (**Figure 1**).

### Antimicrobial Susceptibility Profile

**FIGURE 2:**
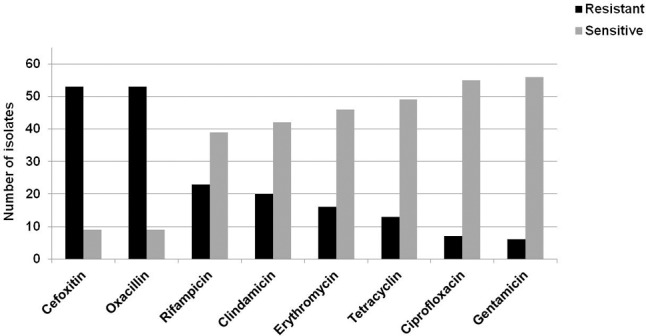
Antibiotic susceptibility profiling of the clinical isolates of CoNS causing bovine mastitis. Antibiotic susceptibility profiling of all the 62 strains was done against oxacillin, clindamycin, erythromycin, gentamicin, ciprofloxacin, tetracycline, and rifampicin. The bars represent the number of isolates sensitive or resistant strain against the respective antibiotic.

A high resistance was observed toward oxacillin 85.5% (53/62, 83.9%) and cefoxitin (53/62, 83.9%) and moderate resistance were seen against rifampicin (23/62, 37.1%), clindamycin (20/62, 32.3%), erythromycin (16/62, 25.8%), and tetracycline (13/62, 20.9%) (**Figure 2**). Resistance against ciprofloxacin (7/62, 11.3%) and gentamycin (6/62, 9.7%) were low, while all strains were susceptible to vancomycin, teicoplanin and linezolid (**Table 2**). Overall, susceptibility profile revealed 45.16% isolates as multidrug resistant (resistant to three or more class of antibiotics), 46.74% as resistant and only 8.06% as pan sensitive (susceptible to all drugs).

**Table 2:** Characterization of the coagulase negative staphylococci clinical isolates causing bovine mastitis.

Name of the Species	Regional Distribution	Number of Isolates	Multidrug Resistant	Resistant	Sensitive	Toxin genes	Virulence genes
*S. sciuri*	Telangana-14	20	10	10	0	*sea, seb*	*Ý-hlg, pvl*
	Andhra Pradesh-6						
*S. haemolyticus*	Telangana-2	13	3	9	1	*sea, seh, etd*	*Ý-hlg, pvl*
	Andhra Pradesh-7						
	Tamil Nadu-4						
*S. chromogenes*	Telangana-4	10	7	1	2	*sea, seh, see*	*Ý-hlg*
	Tamil Nadu-6						
*S. saprophyticus*	Telangana-6	6	1	5	0	*sea, seh*	*Ý-hlg*
*S. xylosus*	Telangana-5	5	5	0	0	*sea*	*Ý-hlg*
*S. simulans*	Tamil Nadu-2	2	2		0	*seh*	*Ý-hlg*
*S. agnetis*	Telangana-1	2	1	1	0	*Etd*	*Ý-hlg*
	Tamil Nadu-1						
*S. epidermidis*	Andhra Pradesh-1	2	1		1	*seh, tsst*	*Ý-hlg*
	Tamil Nadu-1						
*S. gallinarum*	Telangana-1	1	1	0	0	*–*	*Ý-hlg, pvl*
	
*S. cohnii*	Telangana-1	1	0	1	0	*Tsst*	*Ý-hlg*
	


*Multidrug resistant refer to CoNS exhibiting resistance against three or more classes of drugs. Resistant refer to CoNS exhibiting resistance against one or two class of drugs. Sensitive refer to CoNS susceptible against all class of drugs.*

Further, multidrug resistance phenotype was observed in species of *S. chromogenes* (*n* = 7), *S. haemolyticus* (*n* = 3), *S. sciuri* (*n* = 10), *S. xylosus* (*n* = 5), *S. saprophyticus* (*n* = 1), *S. gallinarum (n* = 1), and *S. epidermisis (n* = 1). Resistance against one or two class of antibiotics was observed in *S. sciuri* (*n* = 10), *S. haemolyticus* (*n* = 9), *S. saprophyticus* (*n* = 5), *S. agnetis* (*n* = 1), *S. chromogenes* (*n* = 1), *S. cohnii* (*n* = 1), *and S. simulans* (*n* = 2). Pan sensitive phenotype was observed for the four species, *S. chromogenes* (*n* = 2), *S. haemolyticus* (*n* = 1), *S. agnetis* (*n* = 1), *and S. epidermidis* (*n* = 1).

### Classification of Oxacillin Susceptible and *mecA* Positive Strain (OS-MRS)

Oxacillin susceptibility determined 53 oxacillin resistant and 9 oxacillin sensitive isolates. Seven out of the nine susceptible isolates contain the *mecA* gene representing (OS-MRS) staphylococci. This characteristic was observed among four different species of CoNS, oxacillin susceptible *mecA* positive *S. chromogenes* (OS-MRSC, *n* = 3), oxacillin susceptible *mecA* positive *S. agnetis* (OS-MRSAg, *n* = 2), oxacillin susceptible *mecA* positive *S. sciuri* (OS-MRSS, *n* = 1), and oxacillin susceptible *mecA* positive *S. epidermidis* (OS-MRSE, *n* = 1) (**Table 3**).

**Table 3:** Characteristics of the seven oxacillin susceptible *mecA* positive Coagulase negative Staphylococci (OS-MRS).

Isolate ID	Species	Region	Oxacillin MIC (μg/ml)	mecA gene	SCC mec type	Multidrug resistant (MDR) / Resistant (R) / Sensitive (S)	Toxin genes	Virulence genes
TG-25	*S. chromogenes*	Telangana	0.31	+	3	R	Nil	Nil
TG-31	*S. agnetis*	Telangana	0.31	+	3	S	etd	Nil
TG-58	*S. sciuri*	Telangana	0.31	+	3	R	sea, seb	Ý-hlg
TG-68	*S. chromogenes*	Telangana	0.31	+	3	S	Nil	Ý-hlg
TN-29	*S. agnetis*	Tamil Nadu	0.31	+	3	S	Nil	Ý-hlg
TN-30	*S. epidermidis*	Tamil Nadu	0.31	+	3	S	seh, tsst	Ý-hlg
TN-37	*S. chromogenes*	Tamil Nadu	0.31	+	3	S	seh	Ý-hlg


*Multidrug Resistant refer to CoNS exhibiting resistance against three or more classes of drugs. Resistant refer to CoNS exhibiting resistance against one or two class of drugs. Sensitive refer to CoNS susceptible against all class of drugs.*

Overall, susceptibility profiling of the OS-MRS isolates revealed three resistant and four pan-sensitive isolates. OS-MRS belonged to Telangana (*n* = 4) and Tamil Nadu (*n* = 3). All OS-MRS isolates belonged to same SCC*mec* type III. Toxin genes found in OS-MRS isolates were *Ý-hlg* (*n* = 7), *seh* (*n* = 3), *sea* (*n* = 1), *etd* (*n* = 1), *tsst* (*n* = 1), and *pvl* (*n* = 1) genes.

However, we also found an isolate belonging to *S. chromogenes* species from Tamil Nadu, which was oxacillin resistant and negative for *mecA* gene.

## Discussion

Bovine mastitis is an economically most dangerous disease impacting the livestock industry with a pooled prevalence rate of 41% (sub-clinical mastitis) and 27% (clinical mastitis) from India (Krishnamoorthy et al., 2017). A total loss of 7165.51 crore rupees was estimated due to mastitis in India (Bansal and Gupta, 2009). A recently published meta-analysis report from India for the period of 2005–2016 indicates *Staphylococcus* sp (45%) to be most predominant mastitis pathogen followed by *E. coli* (14%) and *Streptococcus* sp (13%) (Krishnamoorthy et al., 2017). MRSA have been widely studied in India whereas reports of CoNS are limited, however, they have emerged as predominant pathogen now across the world (Pyörälä and Taponen, 2009). In the current study, we have characterized 62 CoNS isolates belonging to 10 different species causing bovine mastitis in India. *S. sciuri* (32.26%), *S. haemolyticus* (20.97%), and *S. chromogenes* (16.13%) were seen as a dominant species causing mastitis which was also observed in a previous finding from India (Preethirani et al., 2015). The oxacillin susceptibility revealed 53 isolates as MRS and 9 isolates as methicillin sensitive staphylococci (MSS). Further, susceptibility profiling against other antibiotics revealed maximum isolates as multidrug resistant or resistant. In the present study, *S. sciuri* and *S. haemolyticus* appeared as the most predominant species in which maximum isolates were methicillin resistant. However, a report from Germany revealed *S. haemolyticus* and *S. epidermidis* as frequently observed MRS species (Fessler et al., 2010).

Methicillin resistance determinant, *mecA* was found in 59 isolates out of which 7 were oxacillin susceptible and are referred as OS-MRS. The OS-MRS belonged to four species: OS-MRSC, OS-MRSAg, OS-MRSS, and OS-MRSE. Recently, the first report of oxacillin susceptible *mecA* positive *S. haemolyticus* isolate has surfaced from China (Li et al., 2015) causing bovine mastitis while from India no incidence of OS-MRS has been reported till date. These OS-MRS isolates were observed from different farms belonging to Telangana and Tamil Nadu. Overall, CoNS with variable traits make it difficult to characterize it as true MRS on the basis of a single test. It demands the need to include both the phenotypic and the genetic test in order to properly screen MRS isolates. Further, treatment of such isolates with β-lactam antibiotics may result in treatment failure leading to the death of the animal and can develop into more resistant population on exposure to antibiotics. In the present study, we also found an isolate, which was *mecA* negative and was oxacillin resistant with a MIC value of 10 μg/ml. A similar finding with nine *mecA* negative and oxacillin resistant CoNS were reported, in which the oxacillin MICs were 0.5 or 1 μg/ml and were determined sensitive using oxacillin or cefoxitin disk diffusion assay (Fessler et al., 2010).

Additionally, we have also screened for toxin genes using PCR in CoNS, which is an important contributing factor in food poisoning. However, to the best of the available knowledge, they are no reports of the toxin genes profiling from CoNS causing bovine mastitis from India. We found enterotoxin genes mainly, the *sea* and the *seh* present in 25.8% and 12.9% of CoNS, respectively, which was in line with the previous finding from Brazil where the prevalence of the *seh* (25.2%) and the *sea* (18.2%) genes was predominant in CoNS isolates from mastitis cases (Mello et al., 2016). Further, studies on CoNS isolated from food products have also revealed the *sea* gene as the most frequent enterotoxin gene present and responsible for outbreaks of food poisoning (Argudín et al., 2010; Rall et al., 2010). In the present study, the *sea* gene was found majorly in clinical isolates of *S. haemolyticus* and *S. sciuri* from all the three states, however, the *seh* gene was found only in isolates of Telangana and Tamil Nadu.

Virulence or the cytotoxin gene; *Ý-hlg* was found in the higher percentage of isolates amounting to 67.7% as compared to the *pvl* gene which was present only in 6.5% of the total population. Presence of *Ý-hlg* gene in the isolates may impart them with increased cytolytic activity. Interestingly, *Ý-hlg* was present in all the 10 different species dominantly in *S. sciuri* and *S. haemolyticus.* The presence of the *pvl* gene in the CoNS isolates indicates a worrisome situation, as these genes impart enhanced virulence capacity and are mostly reported in CoNS isolates of human origin (Baba et al., 2002; Mistry et al., 2016). The data on the prevalence of the *pvl* gene in animal origin CoNS isolates is limited, we found 4/62 isolates positive for the *pvl* gene which is similar to a recent study in which they found 3/81 cows harboring the *pvl* gene, although all isolates were methicillin sensitive which was not observed in our study (Unal and Cinar, 2012). We also found two isolates to be positive for the *tsst* gene, which is not reported earlier in CoNS of animal origin. Therefore, it is crucial to screen for these genes, which make them more potent threat and are only well studied in *S. aureus* causing human and animal infection in comparison to CoNS.

## Conclusion

The present report features the emergence of CoNS isolates with variable phenotypes and genotypes, especially the OS-MRS causing bovine mastitis in India. The findings also stressed the need to include both the susceptibility and genetic tests in order to properly characterize and differentiate among MRS and OS-MRS isolates for correct treatment regime. Further, the majority of multidrug resistant CoNS isolates contain virulence genes, which may result in more serious infection state and a worrisome condition for the veterinarian in the field with limited treatment options. Therefore, studies involving screening of the prevailing population is very crucial, as it will provide us with the updated status regarding the emergence of any genotypic or phenotypic variable antibiotic resistant strain. In future, we plan to investigate the resistance mechanism of such emerging variable strains, which will enhance our understanding and provide with useful insight to design new treatment or diagnostic approaches.

## Author Contributions

VB conceived and designed the experiment. SM, HM, and VB performed experiment. VB and PS data analysis. VB, PS, and RS provided reagents and sample. VB and PS drafted the manuscript. SC and VB manuscript editing and final preparation.

## Conflict of Interest Statement

The authors declare that the research was conducted in the absence of any commercial or financial relationships that could be construed as a potential conflict of interest.
